# Characteristics of hospitalized children with immunoglobulin A vasculitis: a 7-year retrospective study of 119,217 pediatric inpatients in China

**DOI:** 10.3389/fped.2026.1965302

**Published:** 2026-10-27

**Authors:** Yue Ma, Xinyu Wang, Ningning Zhang, Chang Liu, Xin Mo, Yue Hou, Qiang He

**Affiliations:** 1 Department of Traditional Chinese Medicine, National Center for Children’s Health, Beijing Children’s Hospital, Capital Medical University, Beijing, China; 2 Big Data Center, National Center for Children’s Health, Beijing Children’s Hospital, Capital Medical University, Beijing, China

**Keywords:** children, China, epidemiology, immunoglobulin A vasculitis, infectious triggers, mycoplasma pneumoniae

## Abstract

**Background:**

Immunoglobulin A vasculitis (IgAV), formerly Henoch-Schonlein purpura (HSP), is the most common systemic vasculitis in children. This study aimed to systematically analyze the demographic, epidemiological, clinical, and infectious characteristics of pediatric IgAV using a large-scale real-world dataset.

**Methods:**

A retrospective study was conducted using inpatient discharge abstracts from Beijing Children's Hospital, Capital Medical University, spanning January 2016 to December 2022. A total of 119,217 pediatric patients (aged 18 years or younger) diagnosed with IgAV were enrolled. Demographic characteristics, clinical classification, infectious triggers, pathogen spectrum, and multisystem comorbidities and complications were systematically analyzed. Statistical analyses were performed using SPSS 26.0. A two-sided *P* < 0.05 was considered statistically significant.

**Results:**

Among 119,217 enrolled patients, the mean age was 7.79 ± 3.17 years with a male-to-female ratio of 1.30:1. School-aged children (50.77%) and Han ethnicity (93.27%) predominated. Southern China (57.91%) and rural areas (56.91%) showed higher case volumes (both *P* < 0.001). The winter-spring period (November-March) concentrated 50.66% of cases (*P* < 0.001). Clinically, unspecified type was most common (57.02%), followed by renal type (20.04%). Renal-type cases showed a significant increasing trend (*P* < 0.001). Co-infections were present in 47.49% of patients. Mycoplasma pneumoniae was the most frequently recorded pathogen (5.82%), compared with Streptococcus pneumoniae (0.32%). Digestive system complications were most prevalent (4.97%).

**Conclusions:**

Pediatric IgAV in this large Chinese inpatient cohort demonstrated male predominance, a winter-spring peak, and an increasing proportion of renal-type classification (from 15.5% to 27.0% over 7 years). Mycoplasma pneumoniae was the most frequently recorded pathogen (5.82%). These findings suggest that systematic multi-pathogen screening (including Mycoplasma pneumoniae) and standardized long-term urinalysis follow-up may be warranted in the management of hospitalized pediatric IgAV patients.

## Highlights

- This 7-year retrospective cohort including 119,217 pediatric participants represents the largest single-center real-world dataset of childhood IgAV published globally to date, providing statistical power to identify epidemiological patterns that may be underappreciated in smaller cohorts.
- We report a notable pathogen spectrum pattern: Mycoplasma pneumoniae was the most frequently recorded pathogen in pediatric IgAV inpatients in China, a finding that contrasts with the traditional emphasis on streptococcal triggers and warrants further mechanistic investigation.
- This study profiles multisystem comorbidities and complications across seven organ systems and documents an observed increase in renal-type classification over time.
- We report stratified epidemiological data covering all seven major administrative regions of China, revealing marked east-west and north-south disparities in IgAV case distribution across China's seven administrative regions, which may reflect geographic differences in climate, population density, and pathogen circulation, though referral patterns likely influence the observed distribution.

## Introduction

Immunoglobulin A vasculitis (IgAV), previously termed Henoch-Schonlein purpura (HSP) and formally renamed IgAV by the 2012 Chapel Hill Consensus Conference, is the most common systemic small-vessel vasculitis affecting children and adolescents worldwide (1–3). The condition is characterized by IgA1-dominant immune complex deposition in small vessels, leading to the classic tetrad of non-thrombocytopenic palpable purpura, arthralgia/arthritis, gastrointestinal involvement, and renal disease (1, 4). Although the majority of pediatric IgAV cases follow a self-limiting course, renal involvement—termed IgAV nephritis (IgAVN; formerly Henoch-Schonlein purpura nephritis, HSPN)—represents the most critical long-term prognostic determinant, with up to 1%–7% of affected children progressing to end-stage renal disease (5, 6).

In recent years, shifts in the spectrum of infectious pathogens have been associated with fluctuations in pediatric IgAV incidence, placing new demands on clinical surveillance (7). Epidemiological studies have reported an annual incidence of 3–27 per 100,000 children, with notable geographic and seasonal variation (2, 8). However, large-scale, systematically analyzed clinical data integrating demographic geography, standardized pathogen testing, and multi-organ involvement metrics remain scarce for Chinese children. Most existing domestic evidence is limited to small single-center cohorts focusing on single organ complications rather than full-spectrum population epidemiology (9, 10).

Drawing upon the research paradigm of a large-scale EHR-based study on pediatric IgA nephropathy (11), this 7-year retrospective real-world study was conducted based on inpatient discharge abstracts from Beijing Children's Hospital, Capital Medical University. A total of 119,217 pediatric IgAV cases were enrolled to systematically analyze the demographic and epidemiological distribution, clinical subtype classification, infectious trigger spectrum, pathogen profile, and multisystem complication characteristics of childhood IgAV.

## Methods

### Study design and subjects

This retrospective study enrolled pediatric patients aged 18 years or younger with a primary diagnosis of IgAV from the inpatient discharge abstracts (the nationally standardized administrative summary of each hospitalization) of Beijing Children's Hospital, Capital Medical University, during the period from January 1, 2016 to December 31, 2022. Primary IgAV was defined according to the EULAR/PRINTO/PRES 2008 classification criteria (3). Patients with secondary IgAV (vasculitis secondary to other systemic diseases, malignancies, or drug exposure) were excluded. To avoid repeated counting bias, only the first hospitalization record was retained for patients with multiple admissions. All identifying information was fully de-identified before data extraction to comply with patient privacy regulations. This study was approved by the Ethics Committee of Beijing Children's Hospital, Capital Medical University [Approval No. (2026)-E-150-R]; the requirement for written informed consent was waived owing to the retrospective, de-identified nature of the administrative data.

### Data collection

The collected clinical indicators were categorized into four dimensions: (1) demographic and epidemiological indicators, including age, gender, ethnicity, geographical region, urban-rural residence, and monthly admission distribution. Seven major administrative geographic regions were defined according to official Chinese geographical division standards: Northeast China, North China, East China, South China, Central China, Northwest China, and Southwest China; (2) clinical classification according to the hospital's internally expanded ICD-10 clinical coding system based on dominant organ manifestations at admission, categorized into six subtypes: abdominal, articular, mixed, skin, renal, and unspecified; (3) infectious trigger data, including infection sites and pathogenic microorganisms, which were derived from co-recorded discharge diagnosis codes in the inpatient discharge abstracts rather than from standardized laboratory testing; and (4) multisystem comorbidities and complications.

### Statistical analysis

All statistical analyses were performed using SPSS 26.0 (IBM Corp., Armonk, NY, USA). Categorical variables were presented as case counts and percentages. Pearson's chi-square test was adopted for intergroup comparisons when cell theoretical frequencies ≥5; Fisher's exact test was applied for sparse subgroups with small sample sizes. Continuous variables were expressed as mean ± standard deviation (SD). Chi-square goodness-of-fit tests were used to assess distribution differences of demographic variables. Linear-by-linear chi-square trend tests were performed to evaluate annual changing trends of clinical subtypes and renal involvement proportions. A two-sided *P* < 0.05 was considered statistically significant. All reported *P* values are two-sided unless otherwise specified. Missing clinical records were excluded from corresponding subgroup analyses only.

## Results

### Demographic and epidemiological characteristics

A total of 119,217 pediatric IgAV patients were enrolled. The annual case volume increased from 18,456 in 2016 to a peak of 21,358 in 2018, followed by a progressive decline to 11,425 in 2022 (*P* *<* 0.001*;* Table 1, Figure 1).

**Table 1:** Baseline demographic and epidemiological characteristics of 119,217 pediatric IgAV patients (2016–2022).

Characteristic	N	%	Distribution
Total patients	119,217	100.00%	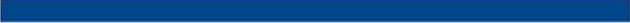
**Year of admission**			
2016	18,456	15.48%	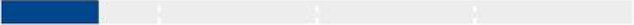
2017	19,696	16.52%	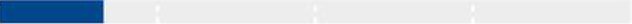
2018	21,358	17.92%	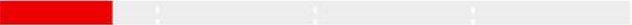
2019	20,942	17.57%	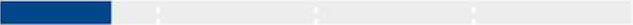
2020	13,736	11.52%	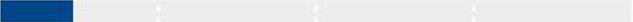
2021	13,604	11.41%	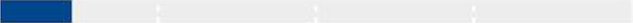
2022	11,425	9.58%	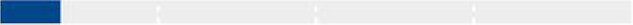
**Age group**			
Infant/toddler	99	0.08%	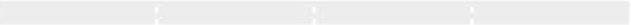
Preschool	7,303	6.13%	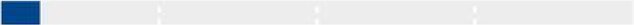
Early school-age	42,072	35.30%	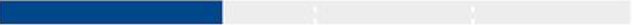
School-age	60,532	50.77%	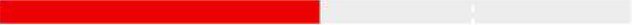
Adolescent	8,142	6.83%	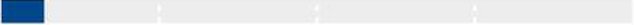
**Gender**			
Male	67,335	56.48%	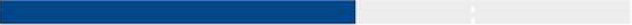
Female	51,882	43.52%	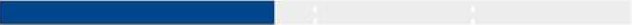
**Ethnicity**			
Han	111,198	93.27%	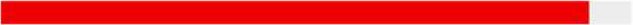
Non-Han	8,019	6.73%	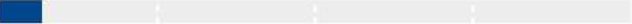
**Geographic region (North/South)**			
South	69,038	57.91%	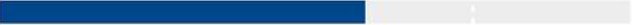
North	50,179	42.09%	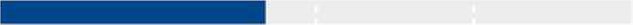
**Urban-rural**			
Urban	50,320	42.21%	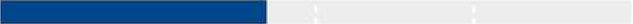
Rural	67,846	56.91%	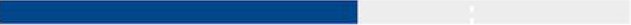
**Seasonal**			
Winter-spring (Nov-Mar)	60,394	50.66%	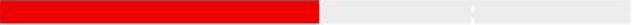
Other months	58,823	49.34%	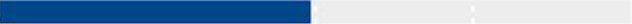

**Figure 1:**
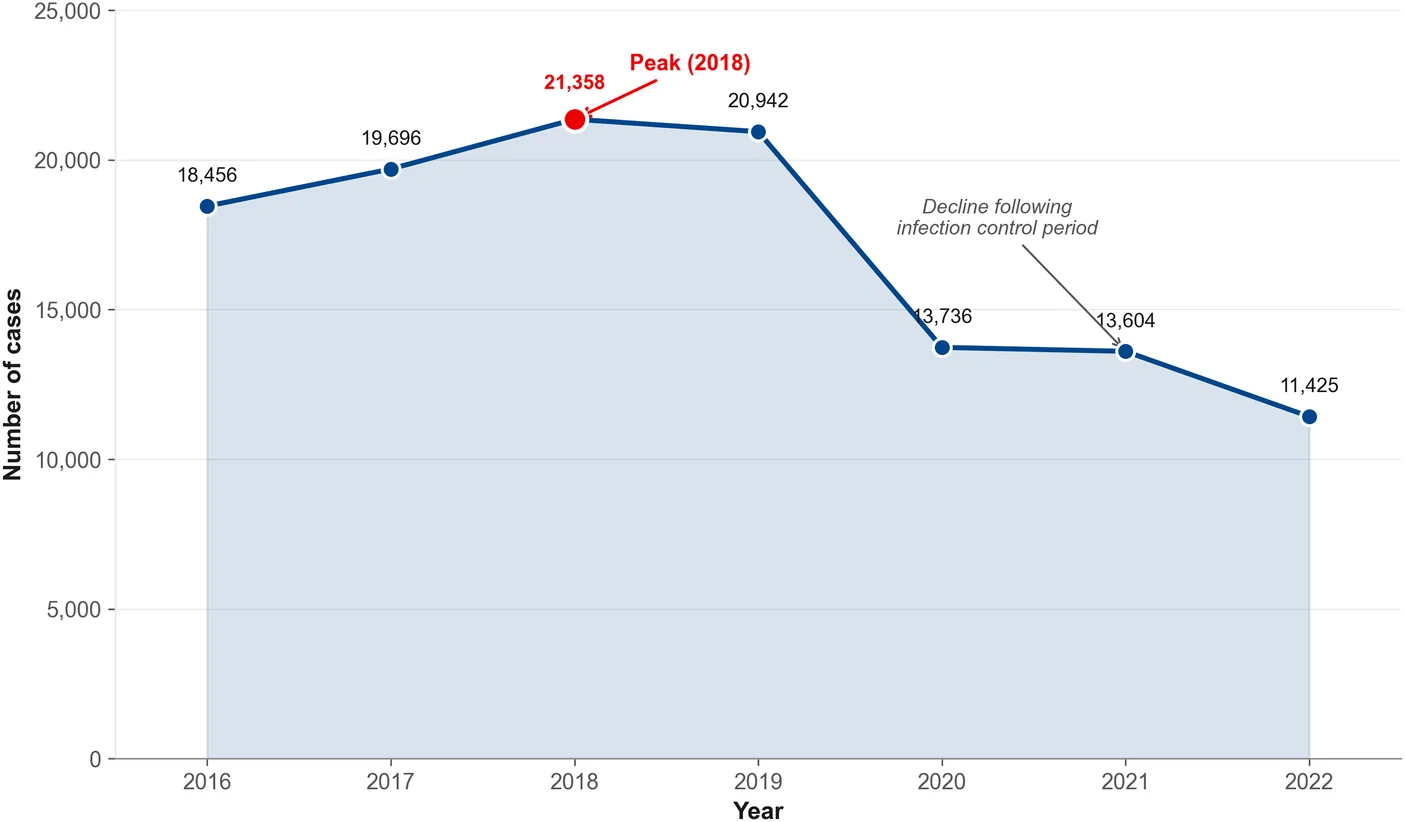
Annual distribution of hospitalized children with IgAV from 2016 to 2022. The *x*-axis represents year of admission; the *y*-axis represents the number of hospitalized cases. The shaded area highlights the peak year and the post-2020 decline.

The mean age was 7.79 ± 3.17 years. School-aged children accounted for the largest proportion (60,532 cases, 50.77%). Infants and toddlers had the lowest morbidity (99 cases, 0.08%; Table 1, Figure 2A). Male predominance was significant (67,335 vs. 51,882, M/F = 1.30:1, *P* < 0.001). Han ethnicity constituted 93.27% (111,198 cases; Table 1). Southern China (57.91%) and rural areas (56.91%) showed higher case volumes (both *P* < 0.001). East China had the highest volume (46,256 cases, 38.80%) vs. Northeast lowest (3,516 cases, 2.95%). Case volume in East China was approximately 13 times higher than that in Northeast China, representing a striking east-west gradient of disease burden (Figure 3). Winter-spring (Nov-Mar) accounted for 50.66% of cases, with the highest number of admissions in December (13,924) and the lowest in July (5,922; *P* < 0.001; Table 1, Figure 2B).

**Figure 2:**
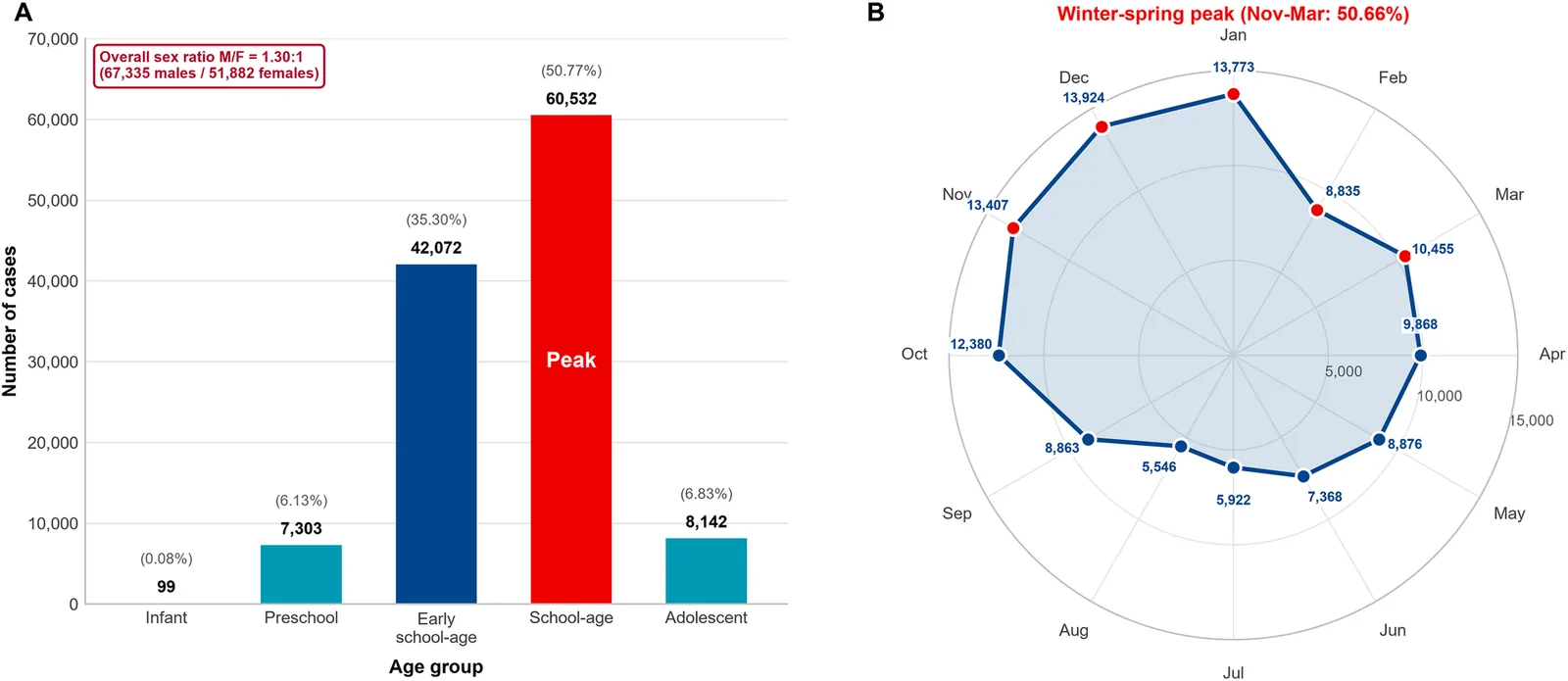
Demographic and seasonal characteristics. **(A)** Age distribution. The *x*-axis represents age groups; the *y*-axis represents the number of cases. The school-age group is highlighted in red. **(B)** Monthly admission distribution presented as a polar radar chart. Each spoke represents a calendar month; the radial axis represents the number of admissions.

**Figure 3:**
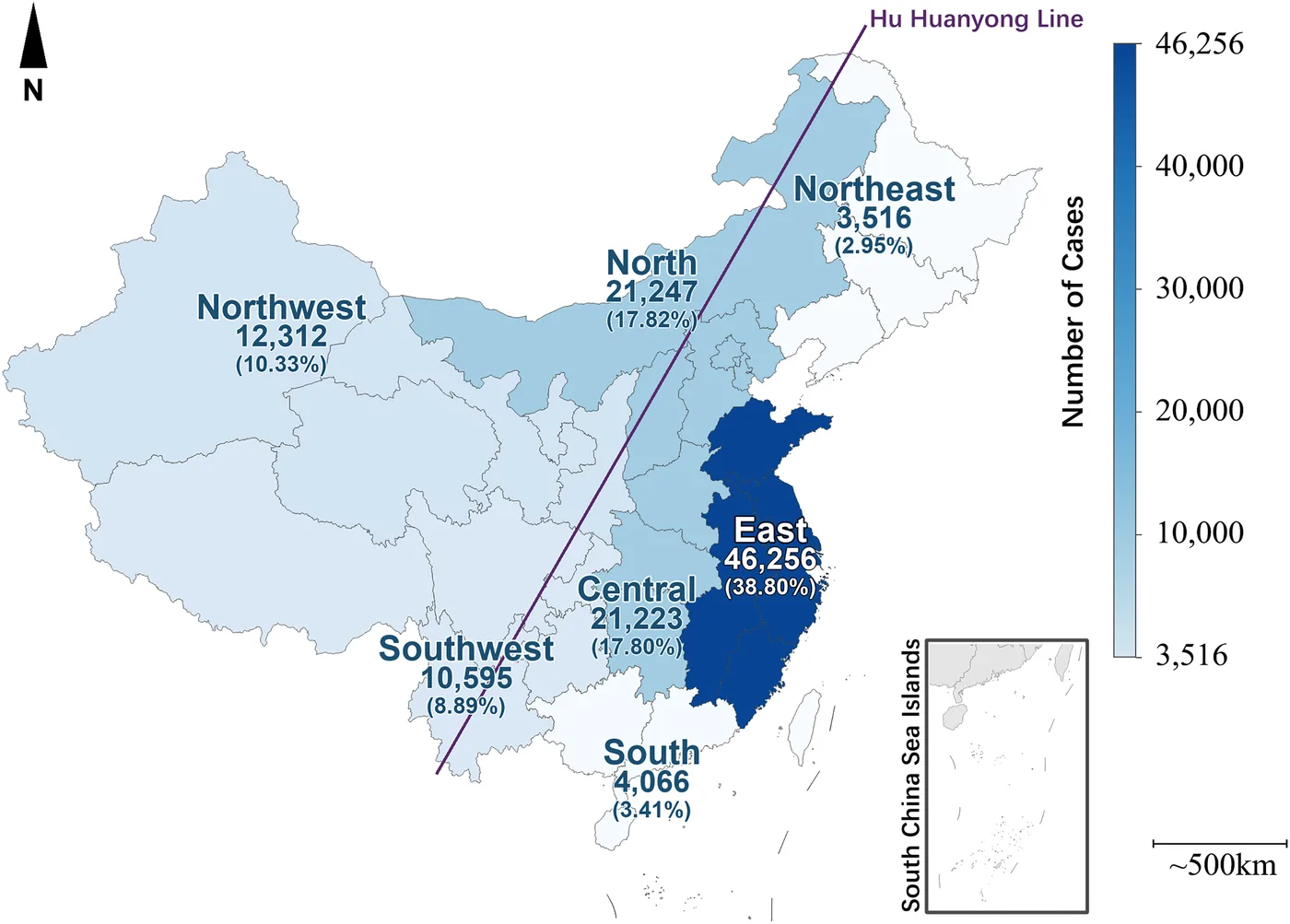
Geographic distribution of pediatric IgAV cases across seven major administrative regions of China. Color intensity represents case volume. The purple dashed line indicates the Hu Huanyong Line (Heihe–Tengchong Line).

### Clinical classification distribution

Distribution (according to hospital ICD-10 expanded coding): unspecified (67,982, 57.02%), renal (23,887, 20.04%), mixed (11,907, 9.99%), abdominal (8,089, 6.79%), skin (4,304, 3.61%), articular (3,048, 2.56%). Renal-type cases increased from 2,857 in 2016 to 3,087 in 2022 (*P* < 0.001), with the proportion rising from 15.5% to 27.0% (*P* < 0.001). Conversely, the skin-only subtype declined from 6.4% (2016) to 3.2% (2022). The annual trends across subtypes are visualized in Figure 4.

**Figure 4:**
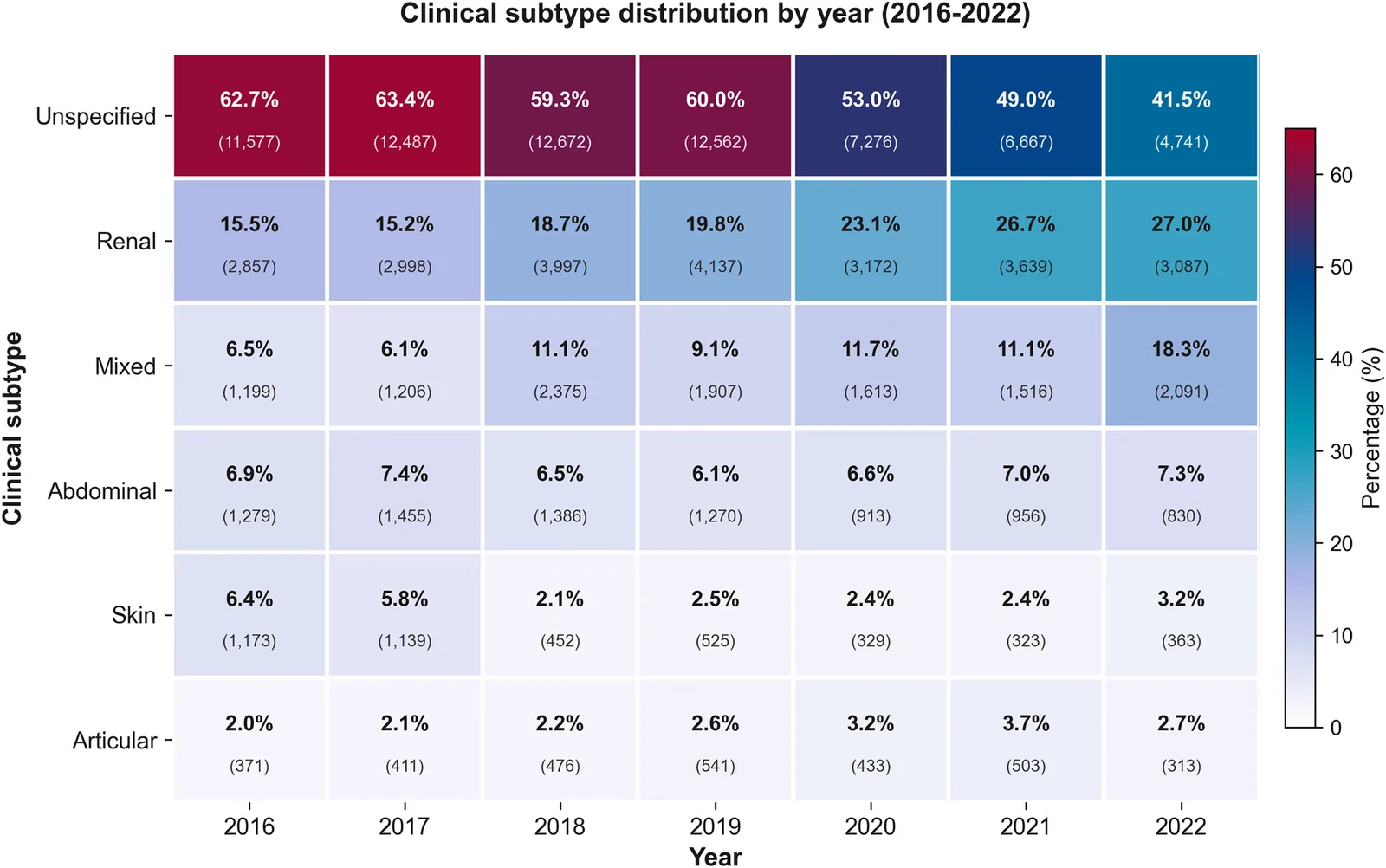
Heatmap visualization of clinical subtype distribution by year (2016–2022). The *x*-axis represents year of admission; the *y*-axis represents clinical subtype. Color gradient reflects the proportion of each subtype within the corresponding year. Cell annotations indicate percentage and absolute case count.

### Infectious triggers and pathogen spectrum

Of 119,217 patients, 56,617 (47.49%) had co-infections. Upper respiratory tract infection dominated (41,712, 73.68%), followed by lower respiratory (13,693, 24.19%), gastrointestinal (1,165, 2.06%), and urinary (47, 0.08%). Infection rate varied by age group (*P* < 0.001). Mycoplasma was most common (6,934, 5.82%) vs. Streptococcus pneumoniae (379, 0.32%) — an 18-fold difference. Helicobacter pylori (HP) and Epstein–Barr virus (EBV) were identified as the second and third most common pathogens, respectively. Both Mycoplasma and S. pneumoniae exhibit winter-spring predominance overlapping with IgAV peak admissions. Infection sites and pathogen spectrum are visualized in Figure 5.

**Figure 5:**
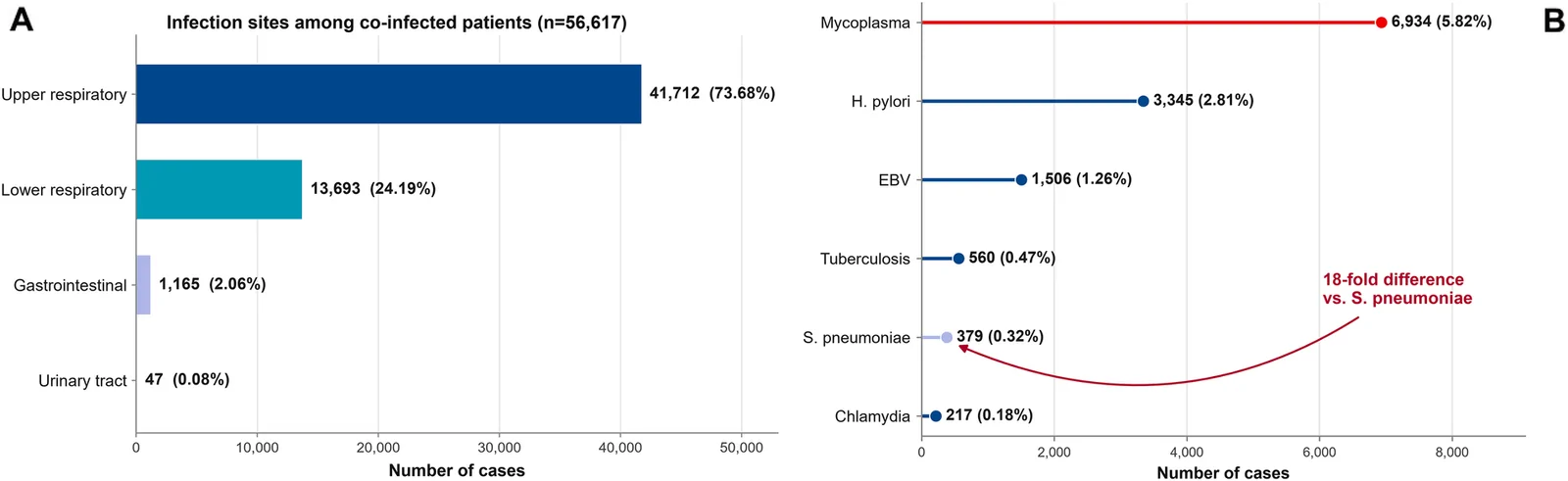
Infectious triggers. **(A)** Infection site distribution. The *x*-axis represents number of cases; the *y*-axis lists infection sites ranked by frequency. **(B)** Pathogen spectrum presented as a lollipop plot. The *x*-axis represents number of recorded cases; the *y*-axis lists pathogen species. Mycoplasma pneumoniae is highlighted in red.

### Multisystem comorbidity and complication distribution

Cardiovascular comorbidities were most frequent (2,153, 1.81%), followed by hematologic (1,863, 1.56%), digestive (1,387, 1.16%), cutaneous (954, 0.80%; mainly urticaria and erythema multiforme), and urogenital (275, 0.23%; mainly urolithiasis and renal dysfunction). Digestive complications were most prevalent (5,922, 4.97%), followed by hematologic (1,219, 1.02%; mainly anemia and hypercoagulable state), cardiovascular (262, 0.22%; mainly hypertension and myocarditis), urogenital (201, 0.17%; mainly orchitis and epididymitis), and neurological (27, 0.02%) including posterior reversible encephalopathy syndrome (PRES). The comparative pattern is visualized in Figure 6.

**Figure 6:**
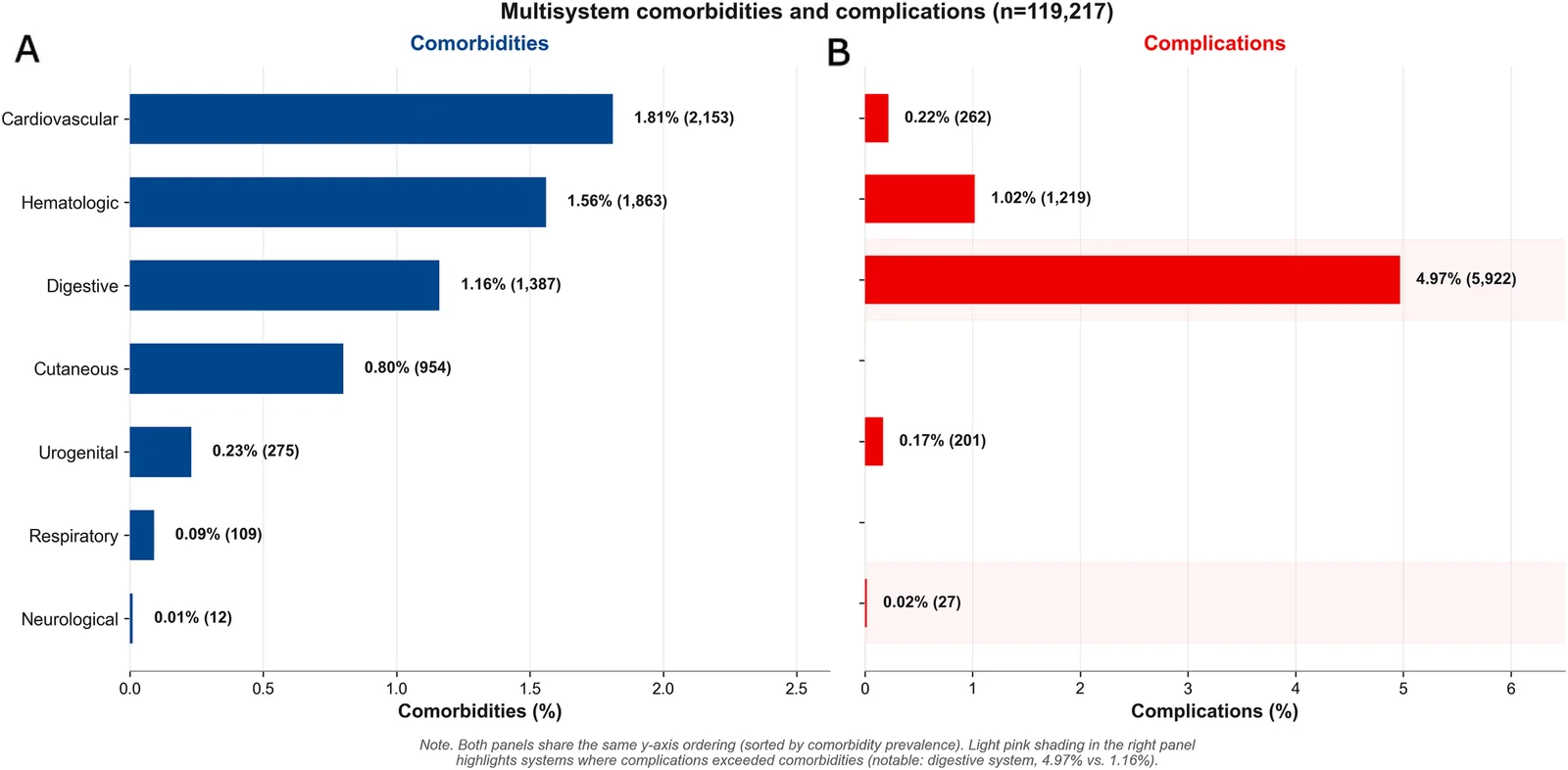
Multisystem comorbidities and complications comparison. **(A)** Comorbidities by organ system. The *x*-axis represents number of cases; the *y*-axis lists organ systems. **(B)** Complications using the same *y*-axis ordering. Light pink shading highlights organ systems where complications exceeded comorbidities.

## Discussion

### Demographic characteristics and geographic distribution

This large-sample 7-year retrospective study systematically analyzed the real-world clinical data of 119,217 pediatric IgAV patients. Its sample size is approximately 8–10 times greater than most prior single-center pediatric IgAV cohorts (12, 13), conferring unique statistical power to identify subtle epidemiological patterns.

The male predominance (M/F = 1.30:1) and peak onset in school-aged children (mean age 7.79 years) align with multiple large-scale epidemiological studies across diverse populations (1, 2). The age group analysis reveals a well-defined pattern — extremely low in infancy, rising steeply in early school age, peaking in mid-childhood, and declining in adolescence that closely parallels the age-dependent trajectory of respiratory infection exposure and school contact density, a pattern consistent with the cumulative infection exposure and immune maturation hypothesis (2). Geographically, the 13-fold East-versus-Northeast disparity is broadly consistent with patterns reported in Chinese multicenter studies (14). The southern predominance (57.91% vs. 42.09%) is consistent with observations from nationwide epidemiological analyses; original meteorological correlation studies have confirmed associations between climatic humidity, temperature and IgAV incidence (15).

### Seasonality and infectious triggers

The winter-spring peak pattern (*χ*^2^ = 9,500.76, df = 11, *P* < 0.001) aligns with previous reports from Korea (8) and nationwide Chinese surveillance (14). The seasonal curve of IgAV precisely overlaps with winter epidemics of influenza, RSV, and Mycoplasma (8, 16), providing temporal evidence for the infection-driven IgAV hypothesis. Notably, Mycoplasma pneumoniae—the dominant pathogen identified in this cohort—also exhibits a well-documented winter-spring predominance in China (16), suggesting that Mycoplasma epidemics may be a key driver of the winter-spring IgAV peak beyond general respiratory viral seasonality. The post-2020 decline in annual case volume is consistent with the reduced circulation of respiratory pathogens during the COVID-19 pandemic, when non-pharmaceutical interventions such as mask-wearing, school closures, and reduced social gatherings suppressed infection exposure—together with changes in healthcare-seeking behavior and hospital admission thresholds during that period.

### Molecular correlates

Previous studies have largely identified Group A beta-hemolytic Streptococcus as the principal infectious trigger of IgAV (4, 17); emerging evidence also highlights Epstein–Barr virus (EBV) as an increasingly recognized viral trigger in pediatric cohorts (18). In this cohort, Mycoplasma pneumoniae (5.82%) was recorded at a substantially higher frequency than Streptococcus pneumoniae (0.32%), representing a notable pattern in the pathogen distribution of this inpatient cohort. It should be acknowledged that Mycoplasma infection shares a winter-spring predominance with IgAV onset, representing a non-negligible confounding factor. However, S. pneumoniae—which shares the same winter-spring epidemic period—was recorded at only 0.32%, suggesting that seasonal overlap alone does not fully account for the observed Mycoplasma predominance. Li et al. (16) have documented shifts in pediatric respiratory pathogen composition based on nationwide surveillance data, closely linked to PCV coverage and antibiotic use patterns. Whether Mycoplasma plays a direct pathogenic role in IgAV or represents a concurrent winter-spring infection cannot be determined from the current data.

At the molecular level, mucosal infections are hypothesized to trigger aberrant production of galactose-deficient IgA1 (Gd-IgA1), leading to immune complex formation, vascular deposition, and complement activation (4, 19). Mycoplasma pneumoniae surface lipoproteins can activate TLR2/TLR6-mediated innate immune signaling, potentially promoting polyclonal B-cell activation and Gd-IgA1 overproduction in genetically susceptible individuals (12, 20). However, as no biomarker data (serum Gd-IgA1, complement profiles, or renal biopsies) were available from this cohort, the relevance of these pathways to our findings remains inferential. Helicobacter pylori and EBV infections may participate in this pathogenic cascade through chronic antigenic stimulation and persistent B-cell activation, respectively (4). These epidemiological observations support the consideration of systematic multi-pathogen screening when evaluating IgAV patients, including Mycoplasma, HP, and EBV.

### Clinical classification and renal involvement

The predominance of the unspecified subtype (57.02%) largely reflects the inherent limitations of ICD-10 coding in capturing nuanced IgAV phenotypes. The observed increase in renal-type classification—from 15.5% (2016) to 27.0% (2022; *P* < 0.001)—should be interpreted with caution. Three overlapping factors may contribute: (i) increased urinalysis screening frequency over time, (ii) improved ICD-10 coding specificity for renal involvement, and (iii) potential genuine epidemiological changes (21, 22). The relative contribution of each factor cannot be disentangled in this retrospective design. This trend should therefore be viewed as a signal warranting clinical vigilance rather than definitive evidence of worsening disease severity. Furthermore, it should be noted that 57.02% of cases were coded as ‘unspecified,’ meaning that subtype-specific analyses—including the observed renal-type trend—are based on the 42.98% of cases with organ-specific coding. This introduces potential selection bias, as physicians may have been more likely to assign organ-specific codes (e.g., ‘renal’) to patients with more severe or clinically apparent involvement, thereby potentially inflating the apparent renal-type proportion. The subtype distribution reported here should therefore be interpreted as a minimum estimate of organ-specific involvement rather than a true population prevalence.

From a molecular perspective, Gd-IgA1-containing immune complexes deposited in the glomerular mesangium are internalized via transferrin receptor (TfR1/CD71)-mediated endocytosis, activating mesangial cells and triggering local complement activation through the lectin and alternative pathways (12, 15, 21, 23). Notably, Koskela et al. demonstrated that even children with clinically mild IgAV may harbor significant glomerular pathology (22). While our cohort lacks histological and biomarker data to verify these mechanisms, the rising IgAVN proportion—whether driven by increased screening or genuine epidemiological changes—reinforces the clinical imperative for standardized long-term urinalysis and renal function follow-up, with lifelong surveillance considered for patients with severe or persistent renal involvement, as long-term risks—including those associated with future pregnancy—have been reported (5, 6, 24–26).

### Multisystem complications

Digestive complications were most prominent (5,922 cases, 4.97%), consistent with previous reports on gastrointestinal involvement in IgAV (27, 28). Cardiac involvement in IgAV is frequently subclinical, and reported frequencies vary widely with the detection strategy used (29). Organ-specific disparities were observed between comorbidity and complication profiles: cardiovascular comorbidities were far more prevalent than cardiovascular complications, whereas digestive complications markedly outnumbered digestive baseline comorbidities, extending current understanding of organ-specific involvement patterns in IgAV.

At the tissue level, Gd-IgA1-containing immune complexes deposited in target organ microvasculature trigger local inflammation through complement activation and neutrophil recruitment; NETosis has been proposed as a potential amplifying mechanism in severe IgAV (30). Posterior reversible encephalopathy syndrome (PRES), documented in 27 patients (0.02%), is associated with acute hypertension-induced cerebrovascular autoregulatory failure and endothelial dysfunction (31); although rare, PRES carries a risk of neurological sequelae, underscoring the necessity of blood pressure monitoring in all hospitalized IgAV patients.

### Study limitations

This study bears several inherent limitations originating from its single-center retrospective inpatient discharge abstract design. First, only inpatient admission records were analyzed, without capturing mild cases managed in outpatient clinics, which may underestimate the overall population burden of IgAV. Second, no laboratory testing of Gd-IgA1, complement components or renal biopsy pathological data was integrated into the EHR dataset; all molecular pathogenic interpretations discussed herein rely on previously published literature rather than direct biomarker measurements from this cohort. Third, infection-related information was derived from discharge diagnosis codes, and the testing practices underlying these codes were not standardized across different admission years, potentially introducing detection bias for infectious triggers. Fourth, ICD-10 administrative coding may lead to minor misclassification of mixed clinical subtypes. Fifth, the relative contributions of increased screening awareness, coding standardization, and genuine disease spectrum change to the observed IgAVN increase cannot be disentangled within this study design. Sixth, long-term follow-up data regarding renal function, proteinuria and chronic renal complications were unavailable; we could not assess the long-term prognostic impact of rising IgAVN incidence in this cohort. Future prospective multicenter studies integrating Gd-IgA1 quantification, complement profiling, standardized pathogen testing, and long-term renal outcomes are warranted.

In summary, this large-scale study based on inpatient discharge abstracts of 119,217 pediatric IgAV inpatients reveals epidemiological patterns consistent with previous reports: male predominance, peak onset in school-aged children, winter-spring seasonality, and notable geographic disparities. Mycoplasma pneumoniae was the most frequently recorded pathogen, and the proportion of renal-type IgAV rose from 15.5% to 27.0% over the 7-year study period.

Based on these findings, we propose the following clinical recommendations: (i) systematic screening for Mycoplasma pneumoniae, Helicobacter pylori, and Epstein–Barr virus should be considered in all hospitalized pediatric IgAV patients, irrespective of respiratory symptoms; (ii) school-aged boys from rural areas presenting during winter-spring months accounted for the highest case volumes in this cohort and may inform clinical suspicion; (iii) standardized urinalysis follow-up should be implemented for IgAV patients, with lifelong surveillance considered for those with severe renal involvement, given the observed increase in renal-type classification; (iv) blood pressure monitoring is warranted to detect rare but serious neurological complications such as posterior reversible encephalopathy syndrome (PRES).

## Data Availability

The original contributions presented in the study are included in the article/Supplementary Material, further inquiries can be directed to the corresponding authors.
